# Maltol Improves APAP-Induced Hepatotoxicity by Inhibiting Oxidative Stress and Inflammation Response via NF-κB and PI3K/Akt Signal Pathways

**DOI:** 10.3390/antiox8090395

**Published:** 2019-09-12

**Authors:** Zi Wang, Weinan Hao, Junnan Hu, Xiaojie Mi, Ye Han, Shen Ren, Shuang Jiang, Yingping Wang, Xindian Li, Wei Li

**Affiliations:** 1 College of Chinese Medicinal Materials, Jilin Agricultural University, Changchun 130118, China; wangzi8020@126.com (Z.W.); 15764380497@163.com (W.H.); junnanhu005@126.com (J.H.); njx006@yahoo.com.cn (X.M.); jiruoxiao@126.com (Y.H.); rs0109@163.com (S.R.); jiangshuang0503@hotmail.com (S.J.); yingpingw@126.com (Y.W.); 2 National & Local Joint Engineering Research Center for Ginseng Breeding and Development, Changchun 130118, China

**Keywords:** maltol, acetaminophen, liver injury, oxidative stress, apoptosis, inflammation response

## Abstract

Maltol, a food-flavoring agent and Maillard reaction product formed during the processing of red ginseng (*Panax ginseng*, C.A. Meyer), has been confirmed to exert a hepatoprotective effect in alcohol-induced oxidative damage in mice. However, its beneficial effects on acetaminophen (APAP)-induced hepatotoxicity and the related molecular mechanisms remain unclear. The purpose of this article was to investigate the protective effect and elucidate the mechanisms of action of maltol on APAP-induced liver injury in vivo. Maltol was administered orally at 50 and 100 mg/kg daily for seven consecutive days, then a single intraperitoneal injection of APAP (250 mg/kg) was performed after the final maltol administration. Liver function, oxidative indices, inflammatory factors—including serum alanine and aspartate aminotransferases (ALT and AST), tumor necrosis factor α (TNF-α), interleukin-1β (IL-1β), liver glutathione (GSH), superoxide dismutase (SOD), malondialdehyde (MDA), cytochrome P450 E1 (CYP2E1) and 4-hydroxynonenal (4-HNE) were measured. Results demonstrated that maltol possessed a protective effect on APAP-induced liver injury. Liver histological changes and Hoechst 33258 staining also provided strong evidence for the protective effect of maltol. Furthermore, a maltol supplement mitigated APAP-induced inflammatory responses by increasing phosphorylated nuclear factor-kappa B (NF-κB), inhibitor kappa B kinase α/β (IKKα/β), and NF-kappa-B inhibitor alpha (IκBα) in NF-κB signal pathways. Immunoblotting results showed that maltol pretreatment downregulated the protein expression levels of the B-cell-lymphoma-2 (Bcl-2) family and caspase and altered the phosphorylation of phosphatidylinositol 3-kinase/protein kinase B (PI3K/Akt) in a dose-dependent manner. In conclusion, our findings clearly demonstrate that maltol exerts a significant liver protection effect, which may partly be ascribed to its anti-inflammatory and anti-apoptotic action via regulation of the PI3K/Akt signaling pathway.

## 1. Introduction

It is well known that drug-induced liver injury (DILI) is a common problem that leads to acute liver failure (ALF) in clinical application and severely affects human health [1]. Acetaminophen (APAP), an antipyretic-analgesic agent, is used in the clinic at therapeutic doses [2]. However, non-intentional misuse may result in hepatic toxicity and high mortality [3]. Initially, cytochrome P450 (CYP) bio-transforms APAP into *N*-acetyl-P-aminophenol (NAPQI), a toxic reactive intermediate, which consumes glutathione (GSH) and leads to mitochondrial dysfunction, oxidative stress, cellular necrosis, and apoptosis and eventually exerts toxic effects on the organism [4,5]. Therefore, seeking novel drugs or supplementary alternatives to prevent APAP-induced liver damage effectively is urgent for researchers.

APAP-mediated hepatotoxicity is closely related to oxidative stress, inflammatory response, and apoptosis. Excessive APAP exposure can cause mitochondrial dysfunction, severe energy debt, and oxidative stress, which induces reactive oxygen species (ROS) and further damage to hepatocytes [6]. Nuclear factor-kappa B (NF-κb) is an important nuclear transport factor and is related to immunoregulation, inflammatory response, and embryo development. Moreover, NF-κB also upregulates death receptors, including tumor necrosis factor-α (TNF-α), FAS (fatty acid synthetase), and the apoptotic proteins of the B-cell-lymphoma-2 (Bcl-2) family [7]. Meanwhile, apoptosis is also regarded as an important research subject in the development of liver diseases. At present, some studies have reported that the phosphatidylinositol 3-kinase/ protein kinase B (PI3K/AKT) signaling pathway is associated with the development of APAP-induced liver injury and early liver regeneration [8]. Based on the above, most researchers have speculated that the inhibition of ROS, apoptosis, and inflammation was a potential target for hepatoprotection.

Maltol (3-hydroxy-2-methyl-4-pyrrolidone) is a flavor enhancer, natural antioxidant, and one of the Maillard reaction products of heated-processed ginseng [9]. Maltol was also found in the roasted Korean ginseng root [10]. In recent years, maltol has been widely used in the fields of catalysis, pharmaceutical preparations, and food chemistry [11,12]. Previous studies have shown that maltol inhibited hexanal oxidation in a dose-dependent manner, and neuroprotective effects of maltol against oxidative stress were also proposed by Kim and Wei et al. [13,14]. Importantly, our previous study confirmed its potent antioxidant properties in TAA (thioacetamide) and alcohol-induced hepatic injury in vivo, which might be attributed to the prevention of lipid peroxidation and alleviation of the inflammation response [15,16,17].

Although maltol was demonstrated to contribute greatly to hepatic organ protection in an alcohol liver injury model, its protective effect on APAP-induced hepatotoxicity has not been further evaluated. Therefore, our aim was to prove the protective effects of maltol on APAP-induced liver injury and explore potential mechanisms of action to develop a reasonable prevention plan for APAP-induced hepatotoxicity.

## 2. Materials and Methods

### 2.1. Chemicals and Reagents

Maltol and APAP were purchased from Sigma-Aldrich (St. Louis, MO, USA). Alanine aminotransferase (ALT), aspartate aminotransferase (AST), GSH, superoxide dismutase (SOD), malondialdehyde (MDA) commercial kits, and haematoxylin and eosin (H&E) dye kits were provided by Nanjing Jiancheng Bioengineering Research Institute (Nanjing, China). Two-site sandwich enzyme-linked immunosorbent assay (ELISA) kits for the detection of mouse TNF-α and IL-1β were purchased from R&D systems (Minneapolis, MN, USA). Antibodies against the rabbit proteins CYP2E1, 4-HNE, Bax, Bcl-2, Bcl-XL, caspase 3, 8, 9, PI3K and p-PI3K, Akt and p-Akt, NF-κB and p-NF-κB, IKKα/β and p-IKKα/β, IκBα and p-IκBα, and β-actin were provided by Cell Signaling Technology (Danvers, MA, USA) and Proteintech (Rosemont, IL, USA). Hoechst 33258 and DyLight 488-SABC immunofluorescence staining kits were provided by Beyotime Institute of Biotechnology (Shanghai, China) and BOSTER Biological Technology (Wuhan, China) dividedly. Other reagents were obtained from Beijing Chemical Factory (Beijing, China).

### 2.2. Animal Experiments

Male ICR mice of 22–25 g body weight were provided by YISI Experimental Animals Co., Ltd. (Changchun, China). Food and water were freely available, and the animals were housed at 23.0 ± 2.0 °C, 50–70% humidity, and light/dark cycle of 12 h. The procedures for all laboratory animals were carried out in strict accordance with the ethical principles adopted in the Laboratory Animal Care and Use Guide (Ministry of Science and Technology of China, 2016). All animal experiments were approved by the Experimental Animal Ethics Committee of Jilin Agricultural University (Ethical Code: ECLA-JLAU-18062). To measure the anti-APAP-induced-hepatotoxicity effect of maltol in mice, the mice were randomly assigned to four groups after one week of adaptation to the environment: a normal group, an APAP (250 mg/kg i.p.) group, and two groups receiving different doses of maltol (50 and 100 mg/kg) (*n* = 8). Maltol was suspended in 0.9% saline. Maltol was administered to mice in two treatment groups for 7 days at doses of 50 mg/kg and 100 mg/kg, respectively. The normal and APAP groups received only 0.9% saline, in the same way. Then, all treatment groups, except the normal group, received a single injection of APAP (250 mg/kg, i.p.) after the final administration to induce acute liver injury. Mice from all groups were euthanized 24 h later. Tissues and blood samples were collected instantly. The serum was stored at −80 °C to determine transaminase after centrifugation (3500 rpm, 10 min, and 4 °C). The liver sections of each group were fixed in formalin for further use.

### 2.3. Analysis of ALT and AST Biochemical Markers

The liver biochemical indicators of serum ALT and AST were measured using commercial detection kits. The samples were transferred to a 96-well plate containing the substrate or a buffer solution and incubated at 37 °C, and the absorbance at 510 nm was measured after adding the developer. All data were expressed as U/L.

### 2.4. Analysis of GSH, SOD, and MDA Oxidative Markers

GSH, SOD, and MDA levels in liver tissues were determined according to commercial reagent methods. The lipid peroxides contained in the sample reacted with thiobarbituric acid (TBA) to form a red mixture. Absorbance at 532 nm was measured. The supernatant of liver tissues was centrifuged at 3500 rpm for 5 min, and then analyzed to determine SOD activity and GSH content.

### 2.5. Analysis of TNF-α and IL-1β Inflammation Markers

After serum samples were obtained, the concentrations of TNF-α and IL-1β were determined using ELISA kits according to the protocols provided by the manufacturer. In brief, prepared reagents, sample standards, and antibodies labeled with enzymes were added, then the reaction was carried out at 37 °C for 1 h. After adding the stopping solution, the absorbance at 450 nm was measured via an ELISA reader (Bio-Rad, Hercules, CA, USA).

### 2.6. Histopathological Examination

For histopathological examination, the liver samples were fixed over 24 h with 10% buffered formaldehyde before paraffin embedding and sectioning into 5 μm thickness. The liver tissues were routinely stained with H&E dye kits (Nanjing Jiancheng Bioengineering Research Institute, Nanjing, China) for conventional morphological evaluation using a light microscope (Olympus BX-60, Olympus Corporation, Tokyo, Japan).

### 2.7. Hoechst 33258 Staining

To observe the nuclear changes of hepatocytes, Hoechst 33258 staining was performed as described previously [18]. The sections were stained with Hoechst 33258 solution (10 μg/mL). UV excitation in a fluorescence microscope allowed us to observe the stained nuclei (Leica TCS SP8, Leica Microsystems, Wetzlar, Germany). The fluorescent intensity was quantified using Image-Pro plus 6.0 software (Media Cybernetics, Rockville, MD, USA).

### 2.8. Immunohistochemistry and Immunofluorescence Staining

As previously described, paraffin sections were deparaffinized and rehydrated prior to dyeing. After antigen retrieval, the slides were incubated with 1% BSA (bovine serum albumin) for 1 h and then with B-associated X (Bax) and Bcl-2 primary antibodies at 4 °C overnight, followed by secondary antibodies for half an hour at room temperature. Positive cells showing a brownish-yellow color in the cytoplasm or nucleus after DAB (diaminobenzidine) and hematoxylin staining were observed [19]. Fluorescence microscopy (Olympus BX-60, Olympus Corporation, Tokyo, Japan) was used for photographing, and positive cells were analyzed by Image-Pro Plus 6.0 software.

Immunofluorescence staining was used to measure CYP2E1 and 4-HNE proteins [20]. Briefly, the sections were incubated with primary antibodies at 4 °C for 12 h, then marked with a secondary antibody for 30 min at room temperature after washing the slides. Finally, the slides were exposed to DyLight 488-SABC. 4, 6 diamidino-2-phenylindole (DAPI) staining used for visualizing the cell nucleui and fluorescence intensities were analyzed by a Leica TCS SP8 microscope.

### 2.9. Western Blot Analysis

Total protein extracts from liver tissues were prepared with RIPA buffer (1:10, *g*/*v*), and a BCA Protein Assay Kit (Beyotime Biotechnology, Shanghai, China) was used for the determination of total protein content. An equal amount of proteins were isolated from the liver tissues and separated by 12% sodium dodecyl sulfate-polyacrylamide gel electrophoresis (SDS-PAGE), then transferred to a polyvinylidene fluoride membrane (PVDF). The membrane was blocked with 5% (*w*/*v*) defatted milk for 2 h and then probed with different primary antibodies at 4 °C for 12 h. After being washed by Tris-buffered saline-Tween (TBS-T), the proteins were incubated for 1.5 h in the presence of secondary antibodies. Protein bands were visualized using Quantity One software (Bio-Rad Laboratories, Hercules, CA, USA).

### 2.10. Statistical Analysis

Results are presented as mean ± standard deviation (S.D.) All samples were tested in triplicate. GraphPad Prism 6.0 (ISI, GraphPad Software, San Diego, CA, USA) was used to analyze the data. ANOVA, followed by the Bonferroni post-test, was used for comparing the differences among groups: *p* < 0.05 or *p* < 0.01 were considered statistically significant.

## 3. Results

### 3.1. Maltol Ameliorated APAP-Induced Hepatic Dysfunction

**Figure 1:**
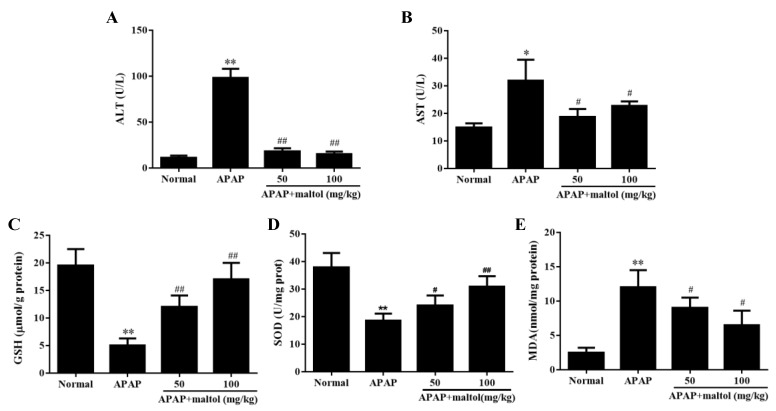
Effects of maltol pretreatment on hepatic dysfunction and histopathological changes caused by an overdose of acetaminophen (APAP). (**A**) serum alanine aminotransferase (ALT) and (**B**) aspartate aminotransferase (AST) activities; (**C**) liver glutathione (GSH) and (**D**) superoxide dismutase (SOD) amount; (**E**) liver malondialdehyde (MDA) content. All data were expressed as mean ± S.D; *n* = 8, * *p* < 0.05, ** *p* < 0.01, vs. normal group; # *p* < 0.05, ## *p* < 0.01 vs. APAP group.

The liver levels of ALT and AST were elevated after APAP (250 mg/kg) injection (*p* < 0.01, *p* < 0.05) compared to those of the normal group, which indicated that hepatocellular damage induced by APAP was successfully established. Supplementation with maltol (50 and 100 mg/kg) for 1 week inhibited the increase in ALT and AST levels after exposure to APAP treatment (*p* < 0.01, *p* < 0.05) (Figure 1A,B).

### 3.2. Maltol Mitigated APAP-Induced Oxidative Stress Injury

APAP-induced oxidative stress injury is associated with the antioxidant defense system. Compared to the normal group, GSH and SOD contents significantly decreased in the APAP group (*p* < 0.01). However, maltol inhibited the depletion of GSH and restored hepatic SOD activity caused by APAP (Figure 1C,D) (*p* < 0.01, *p* < 0.05). In addition, maltol could also block the APAP-induced increase of MDA level in the liver (Figure 1E) (*p* < 0.05). These results clearly demonstrated that maltol reduced the oxidative stress injury caused by APAP.

**Figure 2:**
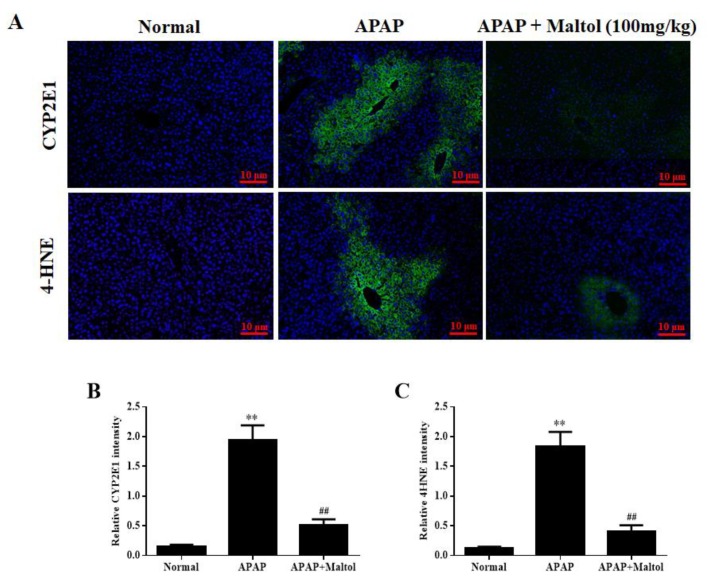
(**A**) Effects of maltol pretreatment on the expression of cytochrome P450 E1 (CYP2E1) and 4-hydroxynonenal (4-HNE) in liver tissues. The expression levels of 4-HNE and CYP2E1 (green) were determined by immunofluorescence (magnification ×200), and nuclear counterstaining (blue) was performed by 4, 6-Diamidino-2-phenylindole (DAPI). Quantitative fluorescence intensities of CYP2E1-positive cells (**B**) and 4-HNE-positive cells (**C**). All data are expressed as mean ± S.D.; *n* = 8. ** *p* < 0.01 vs. normal group; ## *p* < 0.01 vs. APAP group.

For further evaluation of the hepatoprotective activity of maltol on APAP-induced oxidative stress during the progress of acute liver injury, immunofluorescence staining was used to determine CYP2E1 and 4-HNE expression levels in liver tissues. The results showed that maltol treatment significantly reversed the over-expression of CYP2E1 and 4-HNE caused by APAP compared to the normal group (*p* < 0.01) (Figure 2). These findings further confirmed that maltol ameliorated the oxidative stress injury induced by an overdose of APAP.

### 3.3. Maltol Mitigated APAP-Induced Liver Histopathological Changes

**Figure 3:**
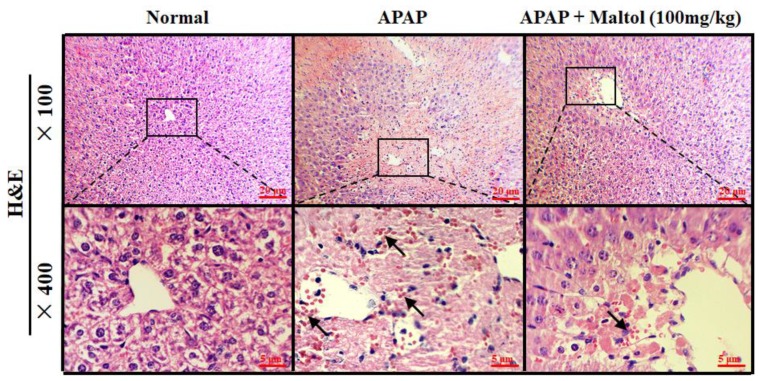
Liver tissue sections were stained with haematoxylin and eosin (H&E) for evaluation of pathological changes.

The results of H&E staining showed that the liver tissue in the normal group was normal and intact, presenting normal cell nuclei and the hepatic central vein. However, severe liver injury characterized by liver structural damage, intrahepatic hemorrhage, and inflammatory infiltration was observed in the APAP group. After maltol treatment for 1 week, inflammation and apoptosis were significantly attenuated (Figure 3).

### 3.4. Maltol Mitigated APAP-Induced Apoptosis

**Figure 4:**
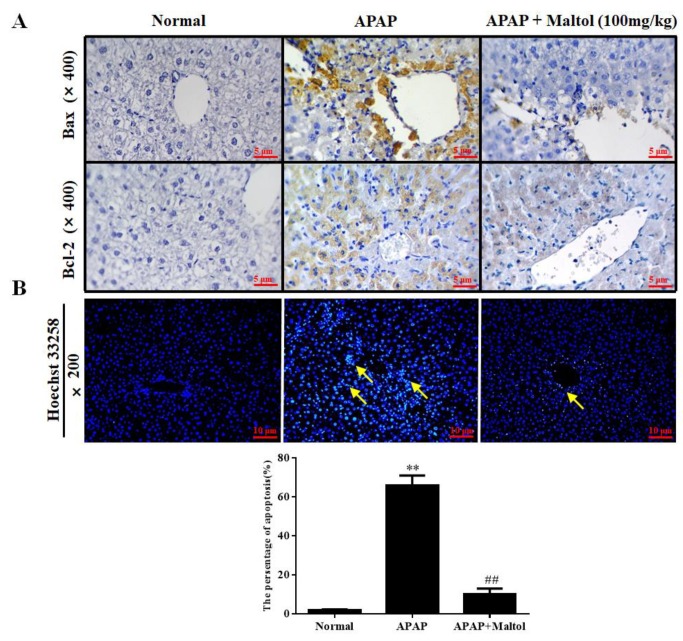
Effects of maltol pretreatment on the expression of B-associated X (Bax) and B-cell-lymphoma-2 (Bcl-2) and Hoechst 33258 staining in liver tissues. (**A**) The protein expression levels of Bax and Bcl-2 were examined by immunohistochemistry in liver tissues (magnification, ×400). (**B**) Hoechst 33258 staining (magnification, ×200). Arrows show necrotic and injured cells. All data are expressed as mean ± S.D., *n* = 8; ** *p* < 0.01 vs. normal group; ## *p* < 0.01 vs. APAP group.

**Figure 5:**
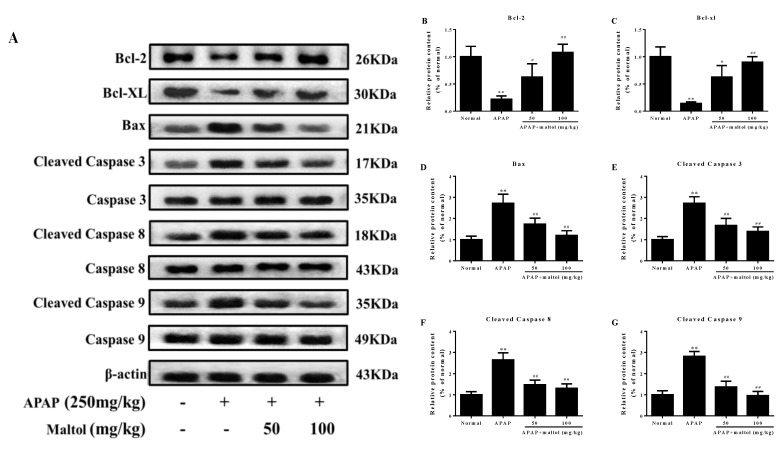
Effects of maltol pretreatment on apoptosis signaling pathways in APAP-triggered acute liver injury (ALI) mice. (**A**) Protein expression levels of Bax, Bcl-2, Bcl-XL, caspase 3, cleaved caspase 3, caspase 8, cleaved caspase 8, caspase 9, and cleaved caspase 9 were measured by western blotting analysis. (**B**–**G**) Quantification of relative protein expression levels was performed by densitometric analysis. All data are expressed as mean ± S.D., *n* = 8; ** *p* < 0.01 vs. normal group; ## *p* < 0.01, # *p* < 0.05 vs. APAP group.

Immunohistochemistry staining, Hoechst 33258 staining, and western bolt analysis were performed to investigate the molecular mechanism of the maltol-mediated beneficial effect against APAP-induced apoptosis by detecting the expression of the apoptotic proteins Bax, Bcl-XL, Bcl-2, caspase 3, 8, 9, and cleaved caspase 3, 8, 9 in liver tissues. Immunohistochemistry staining results demonstrated that APAP exposure caused hepatic cell apoptosis, as indicated by the higher Bax and lower Bcl-2 levels. Nevertheless, maltol could significantly mitigate these changes (Figure 4A). Hoechst 33258 staining supported the above results (Figure 4B). The western bolt analysis results showed that APAP injection markedly increased hepatic Bax and cleaved caspase 3, 8, 9 and decreased Bcl-XL and Bcl-2 levels (*p* < 0.01). Oppositely, apoptosis could be attenuated by maltol (Figure 5) (*p* < 0.01, *p* < 0.05). All results showed that maltol pretreatment dramatically prevented hepatic caspase-mediated apoptosis.

### 3.5. Maltol Mitigated APAP-Induced Inflammatory Responses

**Figure 6:**
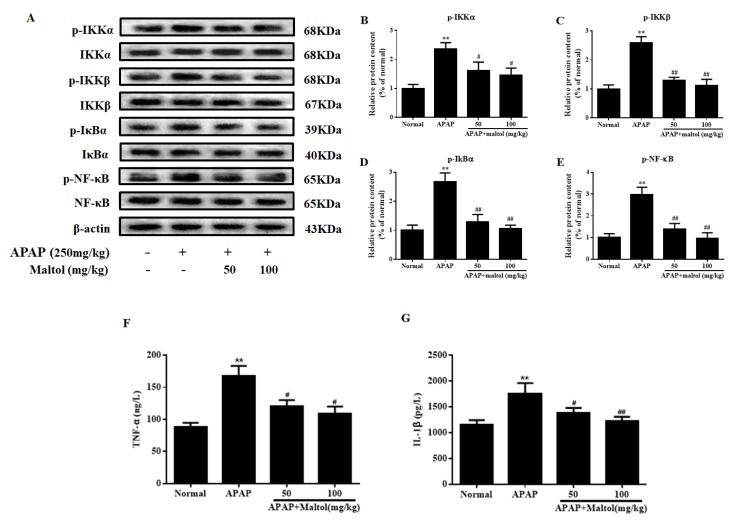
Effects of maltol pretreatment on APAP-induced activation of the nuclear factor-kappa B (NF-κB) signaling pathway in ALI mice. (**A**) Protein expression of phosphorylated and total inhibitor kappa B kinase α/β (IKKα/β), NF-kappa-B inhibitor alpha (IκBα) and NF-κB were measured by western blotting, and β-actin protein was used as a loading control. (**B**–**E**) The relative protein expression levels were quantified by densitometric analysis. The levels of (**F**) tumor necrosis factor α (TNF-α) and (**G**) interleukin-1β (IL-1β) in the serum of mice. Data are expressed as mean ± S.D., *n* = 8; ** *p* < 0.01 vs. normal group; ## *p* < 0.01, # *p* < 0.05 vs. APAP group.

APAP induced a series of inflammatory changes that mediated liver injury. Therefore, a western blot was also used to analyze the anti-inflammatory effects of maltol on APAP-activated NF-κB signal pathway. As shown in Figure 6A–E, APAP resulted in evidently higher levels of phosphorylated NF-κB. The upstream regulators, IKKα/β and IκBα, were also upregulated (*p* < 0.01). However, maltol treatment (50 and 100 mg/kg) prominently suppressed the release of NF-κB phosphorylation and blocked IKKα/β and IκBα phosphorylation (*p* < 0.01, *p* < 0.05), indicating that maltol prevented APAP-triggered inflammatory reaction partly via inhibiting the NF-κB pathway.

In addition, TNF-α and IL-1β are two key proinflammatory cytokines involved in the above progression. In our present study, as shown in Figure 6F,G, APAP injection caused a dramatic increase in the serum levels of TNF-α and IL-1β compared to those in the normal group (*p* < 0.01). However, pretreatment with maltol significantly inhibited the overproduction of TNF-α and IL-1β (*p* < 0.01, *p* < 0.05).

### 3.6. Maltol Regulated the PI3K/Akt Signaling Pathway

**Figure 7:**
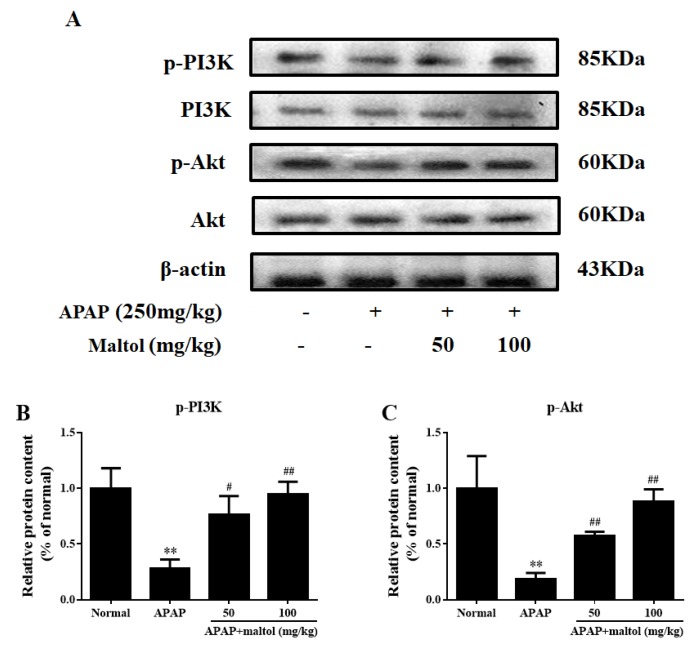
Effects of maltol pretreatment on the phosphatidylinositol 3-kinase/protein kinase B (PI3K/Akt) signaling pathway against APAP-induced liver injury. (**A**) The protein expression levels of phosphorylated and total PI3K and Akt were measured by western blotting with specific primary antibodies, and β-actin protein was used as a loading control. (**B**,**C**) Quantification of relative protein expression levels was performed by densitometric analysis. All data are expressed as mean ± S.D., *n* = 8. ** *p* < 0.01 vs normal group; ## *p* < 0.01, # *p* < 0.05 vs. APAP group.

In order to explore the protective role of the PI3K/Akt signaling pathway, we investigated the effects of maltol on the protein molecules in this signal pathway. From the results of the western bolt analysis, we found clearly that a single exposure to APAP decreased PI3K level and Akt phosphorylation (*p* < 0.01), which is consistent with our previous study [21]. However, maltol could reverse these changes in a dose-independent manner (Figure 7) (*p* < 0.01, *p* < 0.05). The above results showed that maltol exerted a potential protective effect by preventing APAP-induced acute liver toxicity at least partially through modulation of the PI3K/Akt signaling pathway.

## 4. Discussion

According to previous reports, APAP is a common harmful agent when misused or ingested in an excess dose [22]. Hepatotoxicity induced by overdose of APAP has become the most common cause of acute liver failure, replacing viral hepatitis in many developed countries [23]. However, the therapeutic options for this kind of liver injury disease are rather limited. A previous study has shown that maltol exerted beneficial anti-oxidative stress and anti-inflammatory actions in vitro [24]. Given that maltol was confirmed to have various medicinal activities, we evaluated whether maltol has a protective effect for APAP hepatotoxicity. Our former work indicated that maltol pretreatment exerted an important potential and beneficial effect on APAP-triggered acute liver injury and found that its molecular mechanisms of action were related to the alteration of oxidative stress-mediated inflammation and apoptosis, partly via regulation of the PI3K/Akt pathway.

Due to the conjugation with APAP metabolic product NAPQI, the GSH antioxidant system is key to decreasing the toxicity caused by APAP, which causes a sharp depletion of GSH content and then results in the necrosis of hepatocytes [25]. In the present study, it was found that excessive APAP could result in hepatic oxidative stress and cellular necrosis through reducing GSH and SOD levels and increasing MDA production, which were significantly reversed by maltol pretreatment for seven days. These results suggest a potential antioxidant capacity of maltol, in agreement with our previous study [17].

Previous studies have confirmed that oxidative stress is the central mediator of APAP-induced hepatotoxicity [26]. APAP-induced liver injury activates biochemical signaling pathways that originate mainly from CYP2E1-mediated formation of the reactive metabolite NAPQI [27]. It is well known that CYP2E1 has the greatest effect on acute hepatotoxicity caused by APAP, which is a potent inducer of 4-HNE lipid peroxide [28]. Therefore, we evaluated the oxidative stress injury caused by an overdose of APAP by analyzing CYP2E1 and 4-HNE expression. Our results showed that maltol pretreatment could effectively suppress APAP-induced CYP2E1 and 4-HNE overexpression.

Apoptosis plays a critical role in the pathology of tissues and presents with morphological and biochemical features such as DNA fragmentation, cell contraction, and Bcl-2 family protein activation [29]. A key step in apoptotic signaling is the mitochondrial release of cytochrome c, which promotes the formation of apoptotic bodies and the activation of caspase 9, followed by caspase 3 [30]. Activated caspase 3 can promote the cleavage of caspase 8 and amplify the pro-apoptotic signaling pathway through mitochondria. Moreover, cleaved downstream targets perpetuate the apoptotic pathway [31]. In addition, the regulatory factors Bcl-2 and Bcl-XL are two anti-apoptotic molecules of the pro-apoptotic protein Bax heterodimer in the mitochondrial outer membrane, which can prevent the permeability of the outer membrane [32]. A recent study related to mitochondria-dependent apoptosis proved that expression/stability of Bcl-2 could result in the release of the cytochrome and in turn, establish caspase-dependent pathways [33]. As expected, our results clearly indicated that the protein expression of cleaved caspase3, 8, 9 and Bax was remarkably inhibited, while that of Bcl-2 and Bcl-XL was enhanced, indicating that maltol exerted a certain anti-apoptosis effect in APAP-caused hepatotoxicity. Additionally, the result obtained from the Hoechst 33258 staining provided further support that APAP exposure led to a high density of apoptosis, whereas pretreatment with maltol significantly reversed the apoptosis in the liver, corroborating that maltol could dramatically inhibit hepatocyte apoptosis.

Oxidative stress can upregulate pro-inflammatory gene expression [34], and then inflammatory cells can trigger ROS overproduction, which would form a vicious circle and trigger the development of liver damage [35]. NF-κB is a major transcription factor, participating in immunity and inflammation processes, regulating apoptotic genes expression, and then causing apoptosis [36]. Previous literature reported that extracellular stimuli induced the rapid phosphorylation of I-κB and lead to the dissociation of NF-κB from I-κB [37]. Subsequently, activated NF-κB caused transcription of some inflammatory genes, including TNF-α and IL-1β [38]. The pro-inflammatory cytokine TNF-α can activate IKK. I-κB is phosphorylated by activated IKK, and subsequently, the inflammatory signal can also further lead to free NF-κB [39]. In our study, maltol was found to inhibit NF-κB activation by restraining the phosphorylation of IKKα, IKKβ, and I-κBα in a dose-dependent manner. Based on a preceding report describing the anti-inflammatory action of maltol [24], this study suggests that maltol could potentially exert a protective mechanism against APAP liver toxicity that might be partly attributed to the blockade of NF-κB signal activation.

**Figure 8:**
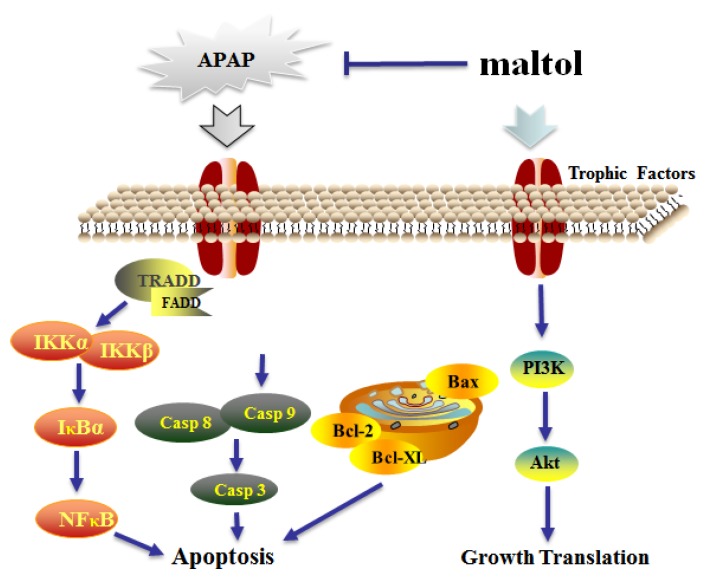
The possible mechanism of action underlying the protective effects of maltol against APAP-induced hepatic injury through inhibition of oxidative stress-mediated activation of the NF-κB pathway and apoptosis and regulation of the PI3K/Akt pathway. Tumor necrosis factor receptor-associated death domain protein (TRADD): Fas-Associated protein with Death Domain (FADD); inhibitor kappa B kinase α (IKKα); inhibitor kappa B kinase β (IKKβ); Caspase 3; Caspase 8; Caspase 9; B-cell-lymphoma-2 (Bcl-2); B-cell-lymphoma-XL (Bcl-XL); B-associated X (Bax); Protein kinase B (Akt); Phosphatidylinositol 3-kinase (PI3K).

PI3K is an intracellular phosphatidylinositol kinase. Akt is a key downstream effector of PI3K, and its anti-apoptotic effect is mainly achieved by phosphorylation of multiple target proteins in downstream pathways [40]. Our previous study proved that inhibited phosphorylation of Akt contributes to APAP-induced liver injury in mice [41]. In this study, maltol was shown to prevent APAP-induced liver injury by activating the PI3K/Akt signaling pathways. A more complete mechanism of maltol anti-APAP hepatotoxicity could include several key signaling pathways (Figure 8).

## 5. Conclusions

In conclusion, the present study proved that maltol exerted a potential therapeutic effect against APAP-induced acute liver injury, which is attributed to its anti-apoptosis, anti-inflammatory, and anti-oxidation activities. The molecular mechanisms of action of maltol involved the suppression of the NF-κB signaling pathway and caspase-dependent cascade and the activation of the PI3K/Akt signaling pathway.

## Abbreviations

The following abbreviations are used in this manuscript:

APAP	Acetaminophen
ALF	Acute Liver Failure
ALT	Alanine aminotransferase
AST	Aspartate aminotransferase
GSH	Glutathione
MDA	Malondialdehyde
ROS	Reactive Oxygen Species
CYP2E1	Cytochrome P450 E1
4-HNE	4-hydroxynonenal
NAPQI	N-acetyl-P-aminophenol
H&E	Hematoxylin and Eosin
NF-κB	Nuclear factor-kappa B
Akt	Protein kinase B
PI3K	Phosphatidylinositol 3-kinase
IKKα	Inhibitor kappa B kinase α
IKKβ	Inhibitor kappa B kinase β
